# Detailed statistical analysis plan for a cluster randomised controlled trial of the Healthy Lifestyles Programme (HeLP), a novel school-based intervention to prevent obesity in school children

**DOI:** 10.1186/s13063-016-1737-y

**Published:** 2016-12-15

**Authors:** Siobhan Creanor, Jenny Lloyd, Melvyn Hillsdon, Sarah Dean, Colin Green, Rod S. Taylor, Emma Ryan, Katrina Wyatt, on behalf of the HeLP Trial Management Group

**Affiliations:** 1 Peninsula Clinical Trials Unit and Medical Statistics Group, Plymouth University Peninsula Schools of Medicine and Dentistry (formerly Peninsula College of Medicine and Dentistry), ITTC Building, Plymouth Science Park, Plymouth, Devon PL6 8BX UK; 2 Institute of Health Services Research, University of Exeter Medical School (formerly Peninsula College of Medicine and Dentistry), South Cloisters, St. Luke’s Campus, Exeter, Devon EX1 2LU UK; 3 Sport and Health Sciences, College of Life and Environmental Sciences, University of Exeter St. Luke’s Campus, Heavitree Road, Exeter, Devon EX1 2LU UK; 4 Isca Academy, Earl Richards Road, Exeter, Devon EX2 6AP UK

**Keywords:** Statistical analysis plan, School-based, Cluster randomised controlled trial, Childhood obesity

## Abstract

**Background:**

The Healthy Lifestyles Programme (HeLP) trial is being conducted to determine whether a novel school-based intervention is effective and cost-effective in preventing obesity in 9–10 year-old children. This article describes the detailed statistical analysis plan for the HeLP trial, including an amendment (and rationale for amendment) made to originally planned sensitivity analyses.

**Methods and design:**

The HeLP trial is a definitive, pragmatic, superiority, cluster randomised controlled trial with two parallel groups and blinded outcome assessment. This update article describes in detail (1) the primary and secondary outcomes, (2) the statistical analysis principles (including which children will be included in each analysis, how the clustered nature of the study design will be accounted for, which covariates will be included in each analysis, how the results will be presented), (3) planned sensitivity analyses, planned subgroup analyses and planned adherence-adjusted analyses for the primary outcome, (4) planned analyses for the secondary outcomes and (e) planned longitudinal analyses.

**Trial registration:**

International Standard Randomised Controlled Trial Number (ISRCTN) register: ISRCTN15811706. Registered on 1 May 2012.

## Background

The World Health Organization regards childhood obesity as one of the most serious global public health challenges for the 21st century. Data from the UK National Child Measurement Programme show that a quarter of children enter primary school overweight or obese and that one third of 10–11-year olds are overweight or obese [1]. The Health Lifestyles Programme (HeLP) was developed with schools, children and their families and aims to motivate and support children and their families in making healthy diet and activity choices [2–5]. The study protocol for the cluster randomised controlled trial of HeLP was published in *Trials* in 2013 and included a brief overview of the statistical analyses [6]. The International Conference on Harmonisation (ICH) guidelines state that primary statistical analyses should be prespecified, to protect from data-driven choice of analyses and selective reporting of outcomes [7]. This update article presents the detailed statistical analysis plan, which was written and approved by the Trial Steering Committee (TSC) in October 2015 (i.e. prior to final database lock), together with agreed amendment to the plan, with the analyses following the updated guidance Consolidated Standards of Reporting Trials (CONSORT) extension statement for cluster randomised trials [8].

## Methods and design

### Brief study overview

This trial of HeLP is a definitive, pragmatic, superiority, cluster randomised controlled trial with two parallel groups and blinded assessment. The study population is 9–10-year old school children attending state primary schools in South West England. All state primary schools with a single year-5 class with more than 20 pupils were eligible, and all pupils from recruited schools were invited to participate.

A total of 32 schools were recruited in 2012 and randomised in a 1:1 ratio to receive either HeLP (intervention) or continue as usual (control), stratified by (1) the proportion of children eligible for free school meals (<19%, ≥19%; which represented the national average of pupils eligible for free school meals at the start of the trial) and (2) number of year-5 classes (one year-5 class, more than one year-5 class). For practical reasons half the schools commenced the study in 2012 (cohort 1) and the other half in 2013 (cohort 2), with equal numbers of control and intervention schools in both cohorts, to facilitate trial delivery. Across the 32 schools, 1324 children were recruited: children and their parents had the option to opt out of the trial before baseline measures were collected. Outcomes were recorded at baseline (before schools were allocated to intervention or control), 12, 18 and 24 months post baseline. Full details of the trial background and rationale, design and sample size calculation have been previously reported [6].

### Intervention

The Healthy Lifestyles Programme is a primary school-based intervention designed to prevent overweight and obesity in children [6]. The intervention has been developed using intervention mapping (with extensive stakeholder involvement) and has been guided by the Information, Motivation, and Behavioural Skills model [9]. HeLP runs over three school terms and includes creating a receptive environment, drama activities, goal setting and reinforcement activities. Full details of the intervention and logic model are published elsewhere [2, 5]. Having successfully completed an exploratory trial with cluster randomisation [3, 4], funding was secured from the National Institute for Health Research (NIHR) Public Health Research programme in March 2012 to run a definitive trial of HeLP.

### Trial objectives

The objectives of this cluster randomised controlled trial are to: (1) assess the effectiveness of HeLP, in children aged 9–10 years, in terms of Body Mass Index (BMI) Standard Deviation Score (SDS) (i.e. at 24 months post baseline), (2) assess the effectiveness of HeLP with respect to a range of secondary outcomes, including further anthropometric measures and classification of weight status at 18 and 24 months and physical activity and food intake at 18 months post baseline, (3) estimate the costs of delivering HeLP and its cost-effectiveness and (4) to conduct a mixed-methods process evaluation and mediational analysis to explore the way the programme worked (that is, how it was delivered, taken up and experienced, and what the behavioural mediators of change are). This article focuses on the analyses planned to address (1) and (2).

### Flow of schools and children

**Fig. 1:**
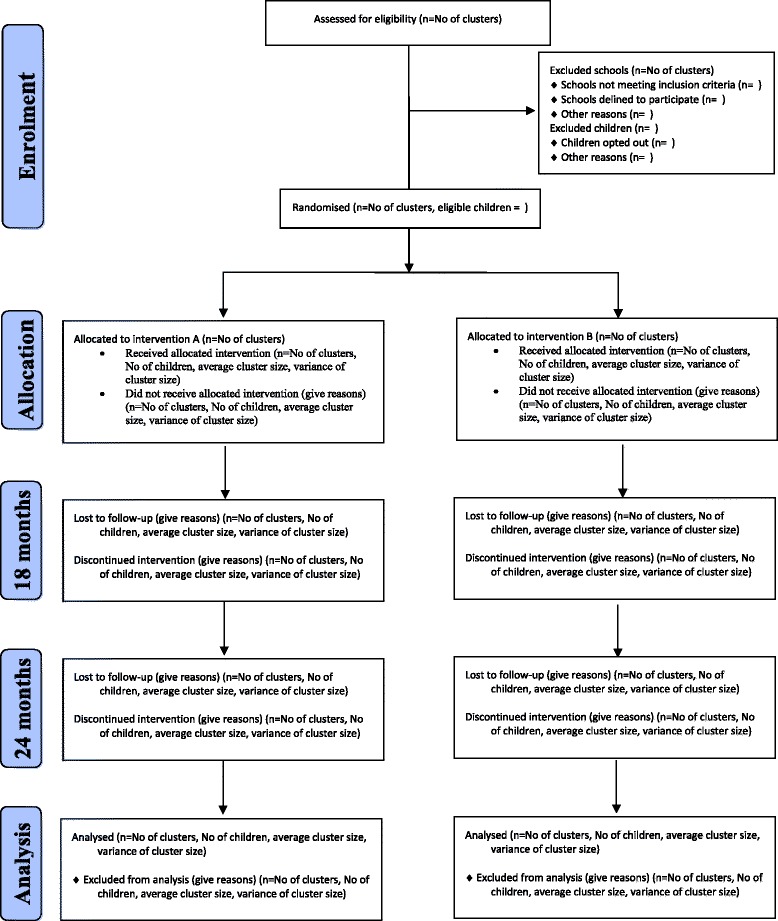
Flow of schools and children through the HeLP trial

The flow of schools and children through the trial will be reported in accordance with the CONSORT extension statement for cluster trials (Fig. 1) [8]. The flow diagram will include the number of eligible and recruited schools, number of eligible and recruited children and then, by allocated group, the number of children who continued through the trial, the number withdrawing at each time point, the number lost to follow-up at each time point and the numbers included in the analysis.

### Withdrawals

In schools allocated to the intervention group, children or their parent/carer could choose to opt out from participating in the collection of outcome measures, whilst participating in the intervention if this was appropriate. Children who discontinue completing the data collection prior to the end of the trial period were withdrawn but will remain in the full analysis population unless they request otherwise. Reasons for withdrawal are documented wherever possible.

### Integrity of data

All outcome data are independently double entered. All inconsistencies between data entries are investigated and any discrepancies discussed with, and resolved by, the trial manager. The integrity of the data has been monitored regularly, with scrutiny of data files for omissions and errors. Range and sense checks will be performed on all variables prior to commencing statistical analyses.

## Outcomes

### Primary outcome

The primary outcome is BMI SDS at 24 months post baseline, measured on the whole cohort of recruited children. BMI SDS indicates how many units (of the standard deviation) a child’s BMI is above or below the average BMI value for their age group and gender. The trial is powered to detect a difference between allocated trial groups in the BMI SDS of 0.25 units at 24 months, with 90% power and a two-sided type 1 error rate of 0.05, assuming the standard deviation of BMI SDS is 1.3 units, the correlation between baseline and 24 months BMI SDS is 0.8 and an intraclass correlation coefficient (ICC) of 0.02 [6]. BMI for each child is calculated from weight and height measurements collected at baseline, 18 and 24 months by assessors blinded to allocated group. Height is measured using a SECA stadiometer (Hamburg, Germany), recorded to an accuracy of 1 mm. Weight is measured using the Tanita Body Composition Analyser SC-330 (Tanita UK Ltd., Middlesex, UK) and recorded to within 0.1 kg and children are asked to take off their shoes and socks [6]. BMI SDS is then derived by converting BMI to centiles using the LMS method and the 1990 BMI UK age and sex thresholds [10, 11].

### Secondary outcomes

1. Anthropometric measures at 18 and 24 months post baseline (whole cohort)
  proportions of children classified as overweight or obese [11]
  waist circumference SDS [12]
  percent body fat SDS [13]
2. Objectively measured physical activity measures at 18 months post baseline (subset of one randomly selected class per school; approximately 67% of whole cohort) [14]
  mean daily time spent sedentary
  mean daily time spent in light physical activity
  mean daily time spent in moderate physical activity
  mean daily time spent in vigorous physical activity
  mean daily time spent in moderate/vigorous physical activity (MVPA)
  mean milli-gravity value (a measure of the average acceleration for each child across the whole observation period)
3. Self-reported food intake at 18 months post baseline (whole cohort) [15]
  mean number of healthy snacks consumed per day
  mean number of energy dense snacks consumed per day
  mean number of positive foods markers consumed per day
  mean number of negative foods markers consumed per day

All the above measures were also captured at baseline.

## General analyses principles

### Participant population

The analysis population will consist of all randomised children excluding: (1) children who left the trial geographical area prior to the collection of baseline measures and (2) withdrawn children who were unwilling for collected data to continue to be used.

The primary analysis for each outcome will be undertaken on an ‘intention-to-treat’ (ITT) basis, i.e. all children with a recorded outcome will be included in the analysis and will be analysed according to the group to which they were allocated [16]. Whilst it is not anticipated that there will be many children who cross-over their trial group (i.e. change from intervention to control, or vice versa), any instances will be documented.

The full analysis population for the primary outcome analysis will consist of all randomised children for whom baseline anthropometric data were collected and for whom 24-month anthropometric data are available. If a child moves to another school within the trial geographical area after baseline measure were collected, they will be invited to continue with the data collection over the remaining period of the trial. As the full analysis population in the primary analysis will exclude a small number of children lost to follow-up (i.e. for whom 24-month BMI data is not available), a sensitivity analysis will be performed to account for all randomised children (see below).

### Levels of confidence and *p* values

Statistical tests and confidence intervals will be two-sided. Between-group comparisons will be calculated and presented with 95% confidence intervals wherever possible. The statistical significance level set will be at the 5% level.

### Unadjusted and adjusted analyses

All comparative analyses will allow for the clustered nature of the data to ensure correct confidence intervals and type I error rates are calculated [17, 18]. As the trial includes a reasonable number of clusters (i.e. 32 schools), the analyses will be based on the individual child-level data, allowing for the clustering between children within the same school, rather than on the cluster-level summarised data, which is appropriate when only a small number of clusters are present [17, 18]. For each outcome, unless otherwise specified, the primary analysis will be the covariate-adjusted analysis, with the statistical models including the two stratification variables (proportion of children eligible for free school meals and number of year-5 classes), cohort, gender and baseline values for the outcome under consideration, where available. Unadjusted between-group differences will be presented for completeness [19].

### Multiple testing

Adjustments will not be made for multiple tests undertaken as the primary outcome of interest is clearly defined. As this is a trial of a complex intervention the secondary outcomes are all potentially of interest and relevance to participants, parents and other stakeholders. Interpretation of the clinical significance of any differences between the two groups will acknowledge the range of variables being measured.

### Missing data

In the event that a child is not available for the collection of outcome measures, up to three additional school visits are organised to try to capture the missing measures. However, even with these additional visits, some loss to follow-up is expected over 24 months. The proportion of children missing each outcome will be summarised for each allocated group and at each time point, with reasons for missing outcomes documented wherever possible. The main analysis of the primary outcome uses the BMI data at 24 months which could be missing for a number of reasons:

1. Parent/carer opts child out of trial before follow-up data collection
2. Child refused to participate in collection of the weight measurement using the Tanita scales
3. Child moved out of the trial geographical area before follow-up data collection
4. Child absent on day of measurement and subsequent follow-up visits
5. Child withdrew from the study

There is no a priori reason to assume that children who are lost to follow-up are missing not at random. Therefore, for the primary analysis, no imputation of missing anthropometric data will be undertaken and this primary outcome analysis will be based on the complete case/observed outcomes dataset [16]. A sensitivity analysis of the primary outcome will account for all children randomised (see below), with missing BMI SDS measures imputed.

In terms of the secondary outcomes, to be included in the physical activity analysis children need to comply with the required minimum continuous wear time of at least 10 h a day for three weekdays and one weekend day. Non-wear will be determined using a published algorithm, details of which can be found elsewhere [20]. In participants who meet the minimum wear time criteria, the data will again be passed over in 60-min rolling windows with 45-min overlap to identify 15-min blocks of non-wear. The 15-min blocks will then imputed based on the average movement recorded in 15-min blocks at the same time of day for the whole monitoring [21]. Any time window with more than 50% non-wear will be treated as missing. For the food intake questionnaire, where a child is missing a subset of the items, the total score will be extrapolated based on the average scores across the four categories (Energy Dense Snacks, Health Snack foods, Negative Food Markers, Positive Food Markers) [4, 15].

### Presentation of comparative analyses

For each of the continuous outcomes (including the primary outcome), the mean and standard deviation for each allocated group will be presented, together with the mean between-group difference, 95% confidence interval for the difference and *p* value. For binary outcomes, the percentage and frequency of children in the outcome category of interest (e.g. percentage overweight/obese) will be presented for each allocated group, along with the odds ratio for the intervention effect, 95% confidence interval for the odds ratio and *p* value. Similarly, for ordinal outcomes, the percentage and frequency of children in each outcome category will be reported for each allocated group, along with the odds ratios, 95% confidence intervals for the odds ratios and *p* value. In addition, the intracluster correlation coefficient will be reported for each outcome, based on the adjusted analyses, together with 95% confidence interval.

## Proposed analyses

### Baseline

Participating schools will be compared to state primary schools in Devon and England at the time of school recruitment into the trial (2012) in terms of (1) percentage of children eligible for free school meals, (2) number of year-5 classes/school size, (3) percentage of children achieving level 4 at Key Stage 2, (4) percentage of pupils with English as an additional language or who are non-white British.

Baseline characteristics, collected at the time of commencing the trial, will be cross-tabulated according to the randomised group to check for appropriate balance and to provide an overview of the study population, both at the school and child levels. At the school level, this will include the percentage of children eligible for free school meals, index of multiple deprivation score for the school’s postcode, number of year-5 classes, and percentage of pupils for whom English is an additional language. At the child level, variables will include gender, age at baseline data collection, individual Index of Multiple Deprivation (IMD) values, baseline measures of all anthropometric measurements, physical activity and food intake. The baseline characteristics of each group will be summarised as the mean, standard deviation and range for continuous, approximately symmetric variables; medians, interquartile range and range for continuous, skewed variables; frequencies and percentages of children/schools in each category for categorical variables.

It is expected that children in both allocated groups will, on average, be similar, given the randomisation procedure. The formal statistical comparison at baseline of randomised groups is not good practice [22] and thus will not be undertaken – only descriptive data, as described above, will be presented. If substantial baseline imbalance between randomised groups is identified in terms of any relevant variables not already being adjusted for in the primary analysis, additional adjusted sensitivity analyses may be performed, to allow for such variable(s), in addition to the prespecified variables for adjustment, to assess the robustness of the primary analysis [19, 22].

### Primary analysis of primary outcome

As described above, the primary analysis of the primary outcome, BMI SDS at 24 months, will follow an intention-to-treat approach, with children analysed according to the trial group to which their school was randomised. Comparisons between the two groups will be implemented using random effects regression, allowing for the clustered nature of the data, and adjusted using the covariates specified above (see ‘Unadjusted and adjusted analyses’ section above).

### Planned and updated sensitivity analyses of primary outcome

A sensitivity analysis will be undertaken after imputing the missing BMI scores, to account for all randomised children. Originally it was anticipated that multiple imputation would be used to impute the missing BMI scores based on the assumption of missing at random. The imputation model was planned to include BMI SDS, gender, cohort and school and any other variables potentially related to missingness, with the imputations performed separately for each allocated group.

During the collection of the final 24-month measures, it was clear that the follow-up rate was very high. After final data cleaning and database lock, we were able to report to our trial management group and TSC that at 24 months we had primary outcome data for 94.4% (1250/1324) of recruited children and 94.8% (1244/1312) of children with baseline BMI SDS. Whilst two thirds of the children with no primary outcome data had moved schools outwith the geographical area, the missing-at-random assumption was less plausible for some of the other children missing at 24 months. Following detailed discussions with our TSC in July 2016, it was agreed that the planned multiple imputation should be replaced by a ‘best case/worst case’ sensitivity analysis. The first of these is based on hypothetically driven assumptions, given the hypothetical preventative nature of the HeLP intervention.

The ‘*best*’ *case scenario* will:

- assume no change between baseline and 24 months in BMI SDS for children allocated to the intervention group, i.e. the baseline BMI SDS value will be carried forward to replace the missing 24-month BMI SDS value
- impute missing 24-month BMI SDS values for children allocated to the control group with their corresponding baseline BMI SDS value plus the (marginal) mean change between baseline and 24 months for the children allocated to the control group with complete baseline and 24-month BMI SDS data.

The ‘*worst*’ *case scenario* will:

- assume that children allocated to the intervention group who were not obese at baseline were obese at the 24-month follow-up: the 24-month BMI SDS value will be set at the Public Health England threshold for obesity (i.e. the 95th percentile; this is 1.645). For children allocated to the intervention group who were obese at baseline, the baseline BMI SDS value will be carried forward to replace the missing BMI SDS value
- impute missing 24-month BMI SDS values for children allocated to the control group with their corresponding baseline BMI SDS value plus the (marginal) mean change between baseline and 24 months for the children allocated to the control group with complete baseline and 24-month BMI SDS data

After imputing the missing 24-month BMI SDS scores for both scenarios, the primary analyses model will then be fitted to the full intention-to-treat datasets to ascertain whether the missing primary outcome data significantly influenced the results of the primary effectiveness analysis.

We anticipate that there will have been only a small number of children who will have ‘switched’ between allocated treatment groups, and hence a ‘per-protocol’ analysis of *actual treatment received* is not likely to be informative. However, the primary analysis intention-to-treat strategy, whilst providing an unbiased estimate of the effect of randomising to intervention or control groups, may underestimate the effect of actually receiving HeLP. Therefore, further exploratory analyses of the primary outcome are planned to estimate the complier average causal effect of treatment (CACE), as a potentially unbiased estimate of receiving HeLP [23]. Compliers can only be observed amongst those randomised to receive HeLP and will be defined as those children who received more than four sessions of drama activities during healthy lifestyles week and who participated in 1: 1 goal setting in phase 3; an indicator variable will be created to identify whether each child randomised to the intervention group complied or not. If there are sufficient numbers within the two categories, compliers and noncompliers within the intervention group will be compared in terms of key baseline characteristics and the estimated CACE between-group difference will be obtained using instrumental variable regression including the same covariates used in the primary analysis, together with randomised group as an instrumental variable for treatment received and including the indicator variable for compliance [23].

### Subgroup analyses of primary outcome

Exploratory analyses of the following possible interactions will be undertaken to assess whether the effect of the HeLP intervention is modified by (1) gender, (2) baseline BMI SDS, (3) number of year-5 classes within school and (4) socioeconomic status. These subgroup analyses will be performed by adding the interaction term between allocated group and the subgroup variable into the regression model. A test of interaction will also be performed to assess whether there is evidence that the effect of the intervention differs across the two cohorts. As the study is not powered for these interaction analyses the results will be treated with caution [24]; given the exploratory nature of these investigations, the emphasis will be on the interpretation of the corresponding confidence intervals for such subgroups. In addition to these subgroup analyses, there is a planned mediational analysis as part of the process evaluation, which will include both moderator and mediator variables.

### Analysis of secondary outcomes

Secondary outcomes will be compared between groups based on the complete data only. Most of the secondary outcomes are of a continuous nature and so comparative analyses will follow the approach detailed above for the primary outcome, using random effects regression, allowing for the clustered nature of the data and including the stratification factors, baseline value of the variable under consideration and gender and cohort. Binary outcomes (such as the proportion of children classified as overweight/obese at 24 months) will be analysed using binary logistic regression, allowing for the clustered nature of the data, and including the stratification factors, baseline BMI SDS, gender and cohort. Ordinal outcomes, e.g. categorisation of weight status, will be similarly analysed using ordinal logistic regression. For all models, corresponding distributional assumptions will be investigated, with consideration given to providing boot-strapped confidence intervals for estimates of between-group differences.

### Longitudinal analysis of anthropometric outcomes

A repeated measures mixed-effects model will also be fitted to all the observed anthropometric measures at baseline, 18 months and 24 months, including effects of time and the interaction term between allocated group and time, to assess whether there is evidence that the effect of the intervention differs across time, taking into account the correlation between measures from the same child, whilst also allowing for the clustered trial design.

### Adverse events

Numbers and percentages of adverse events and serious adverse events will be cross-tabulated with allocated group and also by the actual group, to account for any children who ‘switched’ between intervention and control schools. If there are sufficient numbers of adverse events, binary logistic regression will be used to estimate the odds ratio for the group effect, together with the corresponding 95% confidence interval and *p* value.

## Discussion

The article reporting the protocol for this cluster randomised trial was submitted in May 2012, prior to schools and children being recruited. That article included a brief outline of the planned statistical analyses, which were subsequently further developed after the start of the trial. This detailed statistical analysis plan was written during the delivery period of the HeLP trial (September 2012 to December 2015) and was approved and signed off by the HeLP TSC in October 2015 prior to final data collection. A minor amendment to the planned sensitivity analyses was made following the meeting of the TSC in July 2016, with the amendment approved by the TSC in September 2016, prior to any sensitivity analyses being undertaken. The full analysis plan (including details of the planned mediational analyses and outline of the economic evaluation) is linked to the trial protocol on the NIHR website (www.nets.nihr.ac.uk/projects/phr/10301001).

The rise in the frequency of prospectively publishing statistical analysis plans has followed on from the increasing numbers of trial protocols being published in peer-reviewed journals. In February 2016, a PubMed search with ‘statistical analysis plan’ in the title identified 53 references: the first two statistical analysis plan articles being published in 2009 [25, 26], rising to 15 such papers in 2013 and 2014, with only a small drop to 13 published statistical analysis plan papers in 2015. However, only three of these articles reported on the analysis plan of a cluster randomised trial [27–29], with one reporting analysis plans for a feasibility study, thus analyses of a different nature. The increasing frequency of publishing both trial protocols and statistical analysis plans has been led by a move to increase transparency and ensure that trial outcomes are not selectively reported. The recently established COMPARE (‘tracking switched outcomes in clinical trials’) project assesses the proportion of prespecified outcomes for a trial which are subsequently reported in the associated trial results paper, together with the number of undeclared, not prespecified outcomes reported [30]. The COMPARE team writes to journal editors with notification not only of discrepancies between prespecified and reported outcomes, but also when trial papers include nonprespecified analytic approaches.

By placing statistical analysis plans in the public domain, prior to final database lock, researchers can ensure that the analyses reported in subsequent results papers were prespecified, particularly with regards to secondary analyses, and not data-driven. This is particularly relevant in instances where there are a number of different statistical methods to analyse an outcome. Whilst it is hoped that results would be robust and give similar conclusions, regardless of the method selected, by prespecifying the method(s) of analysis, trialists are prevented from employing different methods of analysis and then reporting only the method which gave the most favourable results.

Finally, we hope that by publishing our detailed statistical analysis plan for a cluster randomised trial of a complex intervention, it may be of use to other teams developing plans for trials of a similar design [31], particularly given that, to date, the vast majority of published articles reporting statistical analysis papers are for hospital-based trials, mainly of medicinal products or devices.

## Trial status

Recruitment of all schools was completed in June 2012, recruitment of all children was completed by September 2013 and the final 24-month follow-up outcomes were collected in December 2015. Final data entry and cleaning is currently underway (February 2016), with final database lock anticipated at the end of February 2016, with the statistical analyses due to commence on time in April 2016. Since submission of the initial version of this paper (February 2016), a minor amendment has been made to the planned sensitivity analyses based on the completeness of the primary outcome data, as reported in the final version of this paper. It is expected that final results will be available in autumn 2016.

## Abbreviations

BMI: Body Mass Index
CACE: Complier average causal effect of treatment
CONSORT: Consolidated Standards of Reporting Trials
HeLP: The Healthy lifestyles programme
ICC: Intracluster correlation coefficient
ICH: International Conference on Harmonisation of technical requirements for registration of pharmaceuticals for human use
IMD: Index of Muliple Deprivation
ITT: Intention-to-treat
MVPA: Moderate/vigorous physical activity
NIHR: National Institute for Health Research
SDS: Standard Deviation Score
TSC: Trial Steering Committee
